# The Epidemiology of Adult Distal Femoral Shaft Fractures in a Central London Major Trauma Centre Over Five Years

**DOI:** 10.2174/1874325001711011277

**Published:** 2017-11-13

**Authors:** Akib Majed Khan, Quen Oat Tang, Dominic Spicer

**Affiliations:** Department of Trauma and Orthopaedics, Imperial College Healthcare NHS Trust, Praed St, London, W2, UK

**Keywords:** Traumatic Knee Injuries, Femoral Fractures, Epidemiology, Distal Femur, Adult, Trauma

## Abstract

**Background:**

Distal femoral fractures account for 3-6% of adult femoral fractures and 0.4% of all fractures and are associated with significant morbidity and mortality rates. As countries develop inter-hospital trauma networks and adapt healthcare policy for an aging population there is growing importance for research within this field.

**Methods:**

Hospital coding and registry records at the central London Major Trauma Center identified 219 patients with distal femoral shaft fractures that occurred between December 2010 and January 2016. CT-Scans were reviewed resulting in exclusion of 73 inappropriately coded, 10 pediatric and 12 periprosthetic cases. Demographics, mechanism of injury, AO/OTA fracture classification and management were analyzed for the remaining 124 patients with 125 fractures. Mann Whitney U and Chi Squared tests were used during analyses.

**Results:**

The cases show bimodal distribution with younger patients being male (median age 65.6) compared to female (median age 71). Injury caused through high-energy mechanisms were more common in men (70.5%) whilst women sustained injuries mainly from low-energy mechanisms (82.7%) (p<0.0001). Majority of fractures were 33-A (52.0%) followed by 33-B (30.4%) and 33-C (17.6%). Ninety-two (73.6%) underwent operative management. The most common operation was locking plates (64.1%) followed by intramedullary nailing (19.6%).

**Interpretation:**

The epidemiology of a rare fracture pattern with variable degrees of complexity is described. A significant correlation between biological sex and mechanism of injury was identified. The fixation technique favored was multidirectional locking plates. Technical requirements for fixation and low prevalence of 33-C fractures warrant consideration of locating treatment at centers with high caseloads and experience.

## 1 INTRODUCTION

Distal femoral fractures represent 3-6% of femoral fractures and 0.4% of all fractures [1, 2]. The literature appreciates a classical bimodal age distribution with younger patients more likely to be male involved in high-energy trauma and older patients are more likely to be female with injury sustained from low-energy etiology such as fall from standing [3]. For the older population, this effect is compounded with the prevalence of osteoporosis [4]. The rapidly ageing population will be responsible for an increased number of fragility fractures affecting the knee [5]. Additionally, the mortality for elderly patients who sustain these injuries may be as high as 18.4%, 39.1% and 48.8% at one, three and five years respectively [6].

The treatment of distal femoral fractures remains a challenge, in particular AO/OTA type B and C [7-9]. Metaphyseal comminution, disruption to the joint surface with often associated small articular segments and sometimes bone loss in open fractures makes it difficult to achieve restoration of joint congruency, stable fixation and early range of movement. Different surgical techniques can be employed depending on patient co-morbidities and pre-morbid function, fracture classification, surgeon choice and patient choice. The range of options include conservative management, screw fixation, pre-contoured locking plate, Intramedullary (IM) nail, external fixation or total knee arthroplasty [10]. Occasionally a combination of the above approaches may be necessary. In the United Kingdom (UK), no nationally accepted guideline exists on the best practice for management of distal femoral fractures. Individual surgeon preference and experience still remains a large factor in determination of treatment strategy.

This paper aims to report the experience of a Central London Major Trauma Center (MTC) in managing adult distal femoral fractures over a five-year period. The complex operative management strategy is also explored.

## 2 METHODS

**Fig. (1):**
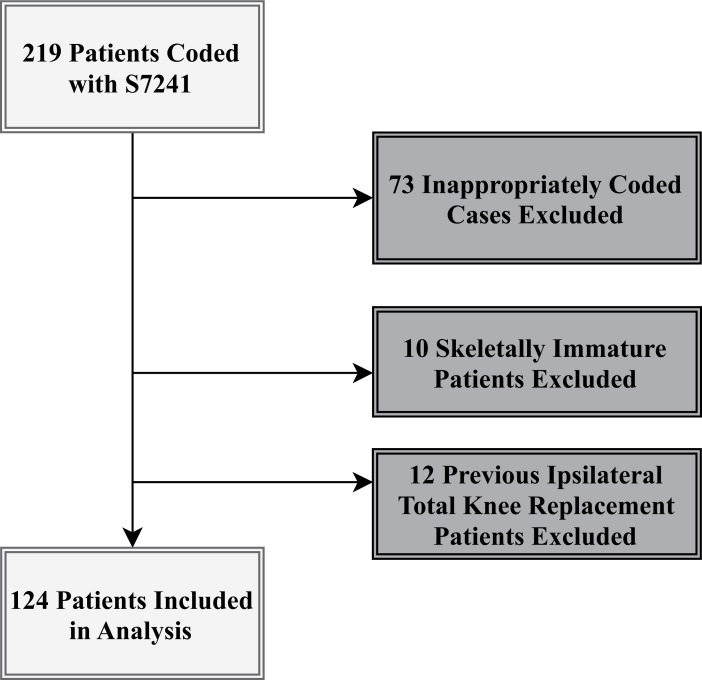
Patient Selection.

This study was conducted in a tertiary referral hospital with MTC status in London, UK. As part of the UK’s MTC network, the hospital provides specialized care and rehabilitation for patients with serious traumatic injuries. Hospital coding records were reviewed for all ICD-10 code ‘S7241 – Unspecified Condyle Fracture of Lower End of Femur’ between the opening of the MTC service on the 1^st^ of December 2010 and the 31^st^ of January 2016, a period of five years and two months. This generated a total of 219 patient cases (Fig. **1**).

Patient cases were cross-referenced with their respective radiographs, the Trauma Audit and Research Network Registry (TARN) and the Trauma and Orthopedic Department electronic notes system to ensure homogeneity between the coding records. Seventy-three inappropriately coded cases (*i.e.* fractures above the metaphyseal flare), 10 skeletally immature patients and 12 patients with previous total knee arthroplasty were excluded.

Data collected for the remaining 124 patients included patient demographics (age at time of injury, sex), mechanism, associated injuries, fracture demographics (side of body, intra-articular extension, AO/OTA fracture classification) and fracture management. The Sex and Gender Equity in Research (SAGER) guidelines were consulted to ensure data collection and design of this study was compliant [11]. Important data was disaggregated according to biologically male and female sex rather than gender. This was decided as biological sex may contribute to earlier loss of bone stock (such as in osteoporosis in females) that may have an implication on the epidemiology of this type of fracture pattern. Data was not disaggregated by gender as discrepancies between biological sex and gender was not felt to be particularly relevant to the study. Radiographs were reviewed by the investigating authors and assigned an AO/OTA classification code.

Non-parametric two-tailed statistical tests were used (Mann Whitney U for univariable data and Chi Squared test for multivariable data). Comparisons were made between the age of patients, mechanism of injury and definitive fracture fixation method.

## 3 RESULTS

### 3.1 Patient Demographics

**Fig. (2):**
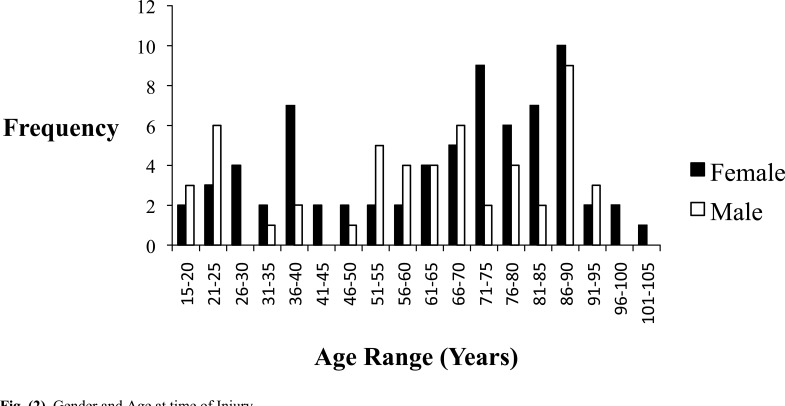
Gender and Age at time of Injury.

Of the 124 patients, 41.9% (n=52) were male (p=0.072) (Table **1**). The mean age was 63 years and the median age was 68 years with a range between 15 years and 101 years. There was a classic bimodal distribution with a higher proportion of patients between 15-40 years and 50-90 years sustaining distal femoral fractures (Fig. **2**). There were a total of 125 fractures with 52.8% (n=66) on the left, 45.6% (n=57) on the right and 0.8% (n=1) bilateral. Although not statistically significant, men were more likely to be younger than women when sustaining these injuries with a median age of 66 years compared with a median age of 71 years (p=0.55). Men had 65.6% (n=21) of the associated injuries cohort.

### 3.2 Mechanism

Mechanism of injury was classified as high energy (fall from >2m, road traffic collision, assault and penetrating injury) or low energy (fall from<2m).

When unspecified injuries were excluded there was a statistically significant difference between sex and mechanism of injury. High-energy trauma was more likely in men (M: 70.5%, n=31 vs. F: 29.5%, n=13) and low-energy trauma more likely in women (M: 17.3%, n=9 vs F: 82.7%, n=43) (p<0.0001).

**Fig. (3):**
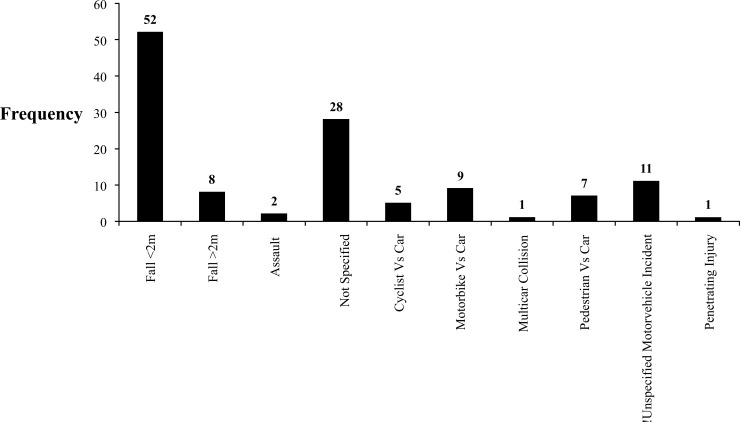
Gross distribution of mechanism causing distal femoral fractures.

The most common mechanism of injury was falling (48.4%, n=60) followed by motor vehicle incidents (26.6%, n=33), assaults (1.6%, n=2) and penetrating injuries (0.8%, n=1). Twenty-eight patients had non-specified injuries or other injuries not coded for within our search parameters (Fig. **3**). Of the 60 patients who fell, the majority did so from ground level (86.7%, n=52). Falling from greater than a two meter height occurred in eight patients (13.3%). The 33 motor vehicle incidents were due to pedestrian vs car (21.2%, n=7), cyclist vs car (15.2%, n=5), motorbike vs. car (27.3%, n=9), multicar collision (3.0%, n=1) or unspecified (33.3%, n=11).

### 3.3 Injury Pattern

**Fig. (4):**
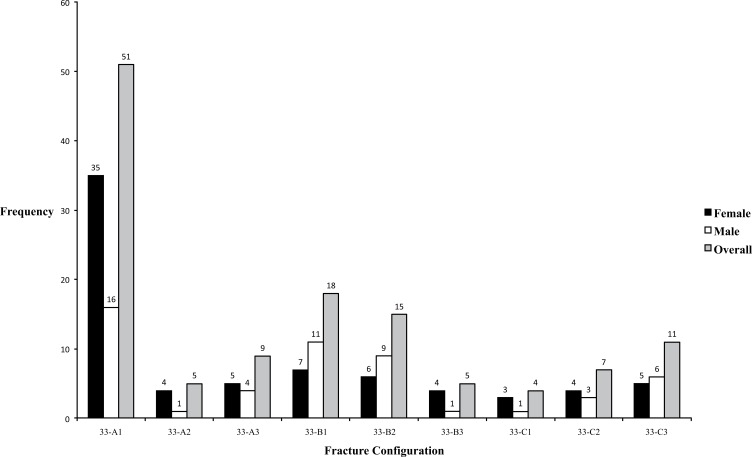
Distribution of Injury Pattern according to AO/OTA Classification System .

Fracture type consisted of 65 (52.0%) type 33-A, 38 (30.4%) type 33-B and 22 (17.6%) type 33-C configurations as seen in Table **1**. Subgroup analysis revealed 33-A1, A2, A3, B1, B2, B3, C1, C2, C3 to comprise 51 (40.8%), 5 (4.0%), 9 (7.2%), 18 (14.4%), 15 (12.0%), 5 (4.0%), 4 (3.2%), 7 (5.6%) and 11 (8.8%) patients respectively (Fig. **4**). Involvement of the knee joint was present in 60 patients (48.0%).

### 3.4 Inpatient Stay

**Fig. (5):**
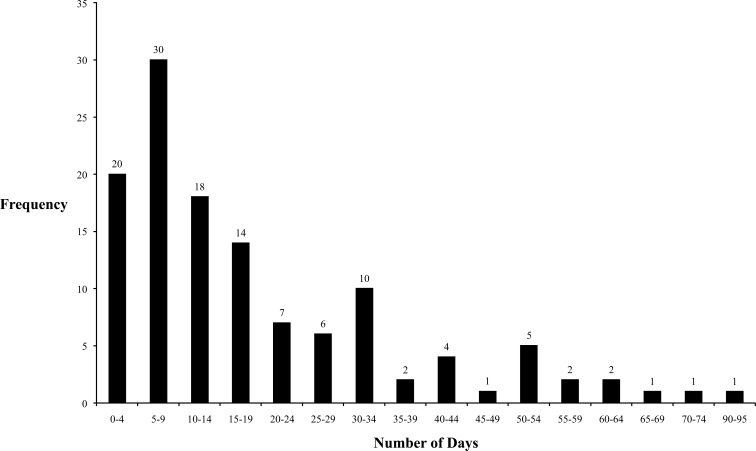
A graph displaying the length of inpatient stay censored at either death or discharge .

The majority of patients had an inpatient stay of less than two weeks when censored at either discharge or death. The longest inpatient stay was 95 days (Fig. **5**).

### 3.5 Associated Injuries

Associated injuries were documented in 32 (25.8%) patients. Thirty (93.8%) of these patients had a second fracture elsewhere.

### 3.6 Fracture Fixation Technique

**Fig. (6):**
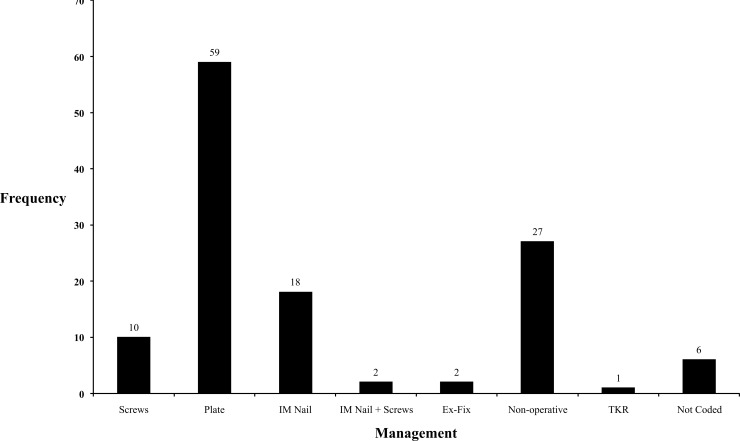
A graph demonstrating the different management options for treating distal femoral fractures.

In total, 92 (73.6%) fractures underwent operative management (Fig. **6**). There were 27 (21.6%) fractures managed conservatively for various reasons including undisplaced stable fractures, extreme frailty and poor pre-morbid function. Six (4.8%) patients had missing operative records. Our experience determined the use of locking plates to be the most common intervention occurring in 59 (64.1%) operations. Other operative approaches include IM nailing (18, 19.6%), screws (10, 10.9%), IM nail and screws (2, 2.2%), external fixation (2, 2.2%) and TKR (1, 1.1%).

## 4 DISCUSSION

### 4.1 Incidence

The MTC in this study is estimated to service a population of around three million in London [12]. This is likely to be an underestimate given the high commuter population and presence of three other MTC hospitals within London. Had the population remained static over a five year and two month period, our study indicates distal femoral fractures that present to MTC status hospitals have an incidence of 8/million/year in an area covering one quarter of Greater London. This figure remains crude. It also does not cover patients who may have presented directly to Trauma Unit hospitals within the catchment area of MTC. It does, however, highlight the relative rarity of such fractures compared to proximal femoral fractures [13].

### 4.2 Age and Sex

Court-Brown and Caesar classified common fractures into eight age-sex distribution curves (A to H) [2]. According to their study, the epidemiology of distal femoral fractures follows curve E (unimodal distribution in elderly females). Our study demonstrates a distribution more akin to curve A. This finding echoes a study by Pietu *et al.* that determined their population of 177 patients with distal femoral fractures at 12 hospitals in France [14]. The mean and median age seen in our experience is similar to that seen in other epidemiological studies [15, 16]. Fifty-eight percent of the patients in our study were female. This is lower than the 73.1% (n=147) females seen in a similar study of 201 distal femoral fractures by Ng *et al.* [17]. Although not discretely reported, Kolmert and Wulff graphically display a much higher proportion of female patients than male patients with this fracture pattern [15]. Baron *et al.* analyzed data from 5% of the U.S. medicare population for fractures that occurred between the years 1986-1990 [18]. They demonstrated that the rate ratio of lower femoral fractures increased between the ages of 70 to 89 in both men and women. The rate ratio was almost double in women of ages 85-89 compared to men within the same age range. Their findings suggest that elderly women in developed countries are more likely to be affected by distal femoral fractures. Our study exclusively disaggregated patients according to biological sex as this approach conforms to previous studies within this domain. Whether gender based differences exist remains a question especially with the use of estrogen and testosterone hormonal therapies for patients identifying to a different gender than their biological sex. Estrogen and testosterone play a role in bone biology and are related to bone stock [19]. However, any potential association between these therapies and the epidemiology of distal femoral shaft fractures is an area for possible future research.

### 4.3 Mechanism

Our study determines that high-energy mechanisms dominate the male population (70.5%, n=31) whilst low-energy trauma dominated the female population (82.7%, n=43). This was statistically significant (p<0.0001). These findings are consistent with reported literature elsewhere [3].

### 4.4 Fracture Pattern

Our study found that fracture patterns 33-A1, 33-B1 and 33-B2 were the most common. Smith *et al.* determined 33-A1 and 33-C1 to be the most common in their study of 105 distal femoral fractures across four MTCs [20]. It is difficult to draw comparisons as patients below the age of 50 years were excluded from their analysis thus different etiology and patient populations can explain the variation. Pietu *et al.* also agrees with type 33-A1 being the most prevalent [14]. Our experience of intra-articular involvement (48.0%) is similar to what has previously been described elsewhere [2, 14, 20].

### 4.5 Operative Management

The majority of distal femoral fractures are managed operatively in adults. Even in elderly patients, non-operative management of displaced fractures is associated with poor outcomes due to increased risk of pressure ulcers, pneumonia, deep vein thrombosis and knee stiffness [21]. Butt *et al.* studied 42 elderly patients with displaced distal femoral fractures [22]. They concluded that operative management provided superior results compared to conservative management. Our figure of 73.6% requiring operation includes patients who had undisplaced fractures or were too systemically unwell to undergo operation. It is similar to the operation rate for distal femoral fractures at four other MTC in the UK [20]. Plate fixation dominated fixation techniques that reflect improved technology and mimics practice at other centers [10].

The link between low-case volume and increased risk of post-operative complications has been shown in the context of total hip arthroplasty [23]. This tentatively supports the case for patients with distal femoral fractures to be cared for in specialized trauma units with higher case loads and more experience. Further study into the epidemiology of distal femoral fractures at trauma units and smaller community hospitals is needed before definitive conclusions can be made.

### 4.6 Fixation Techniques

**Fig. (7):**
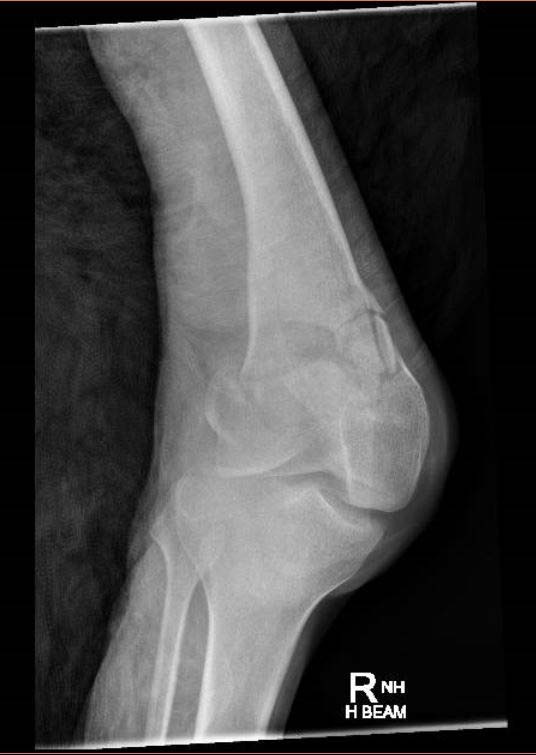
Use of plain radiographs to determine fracture configuration, size of bony comminution and bone stock; Anteroposterior Radiograph.

**Fig. (8):**
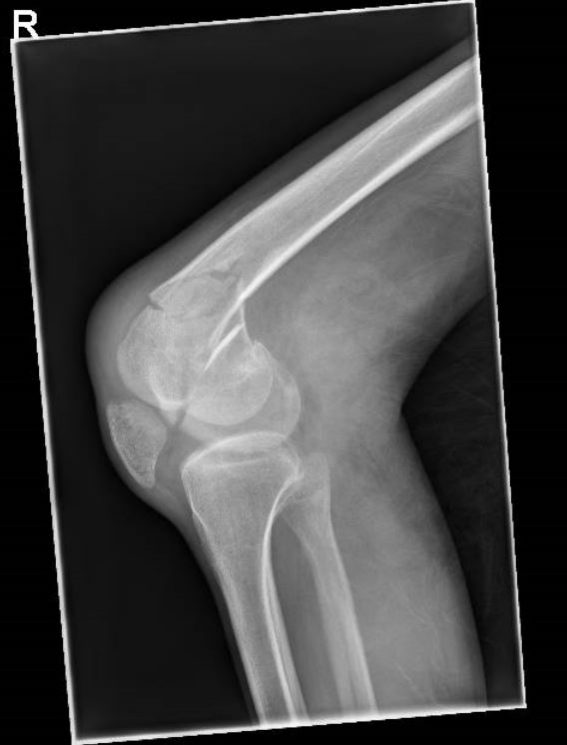
Use of plain radiographs to determine fracture configuration, size of bony comminution and bone stock. Lateral Radiograph.

Once operative management has been decided, obtaining the correct imaging is crucial. Initial anteroposterior (AP) and lateral radiographs of the knee and distal femur are important and provides valuable information about the injury (Figs. **7** and **8**). Fractures around the distal femur are typically shortened and rotated and therefore traction radiographs can be helpful. High-energy injuries mandate radiographs of the pelvis and ipsilateral hip in order to exclude the presence of fractures proximal to the knee.

**Fig. (9):**
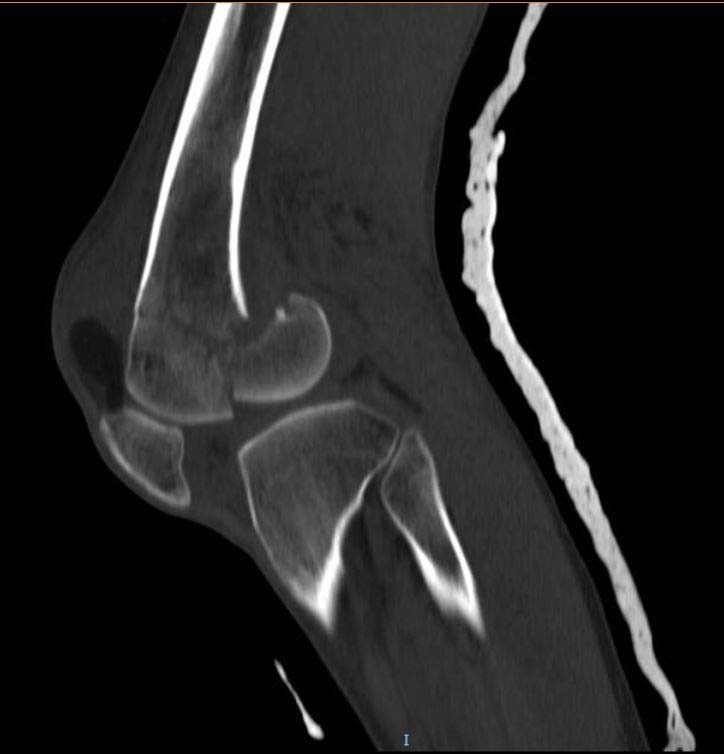
CT Scan in Sagittal Section showing a coronal plane fracture of distal femur (Hoffa Fracture).

**Fig. (10):**
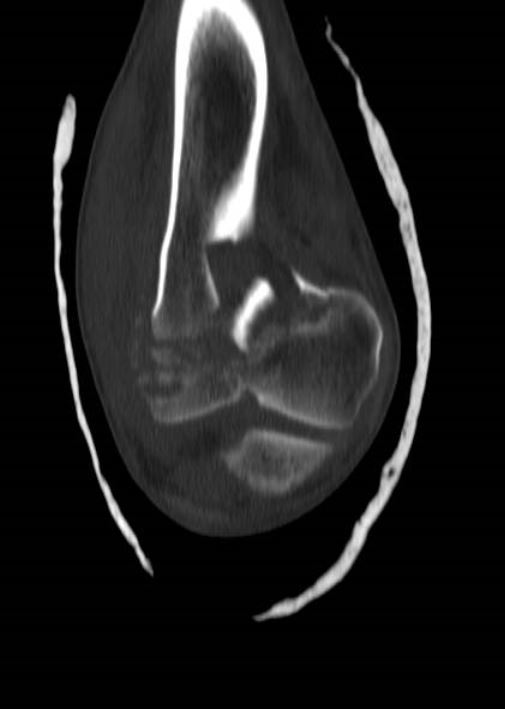
Use of CT Scan to determine fracture configuration, size of bony comminution and bone stock; Coronal Section.

**Fig. (11):**
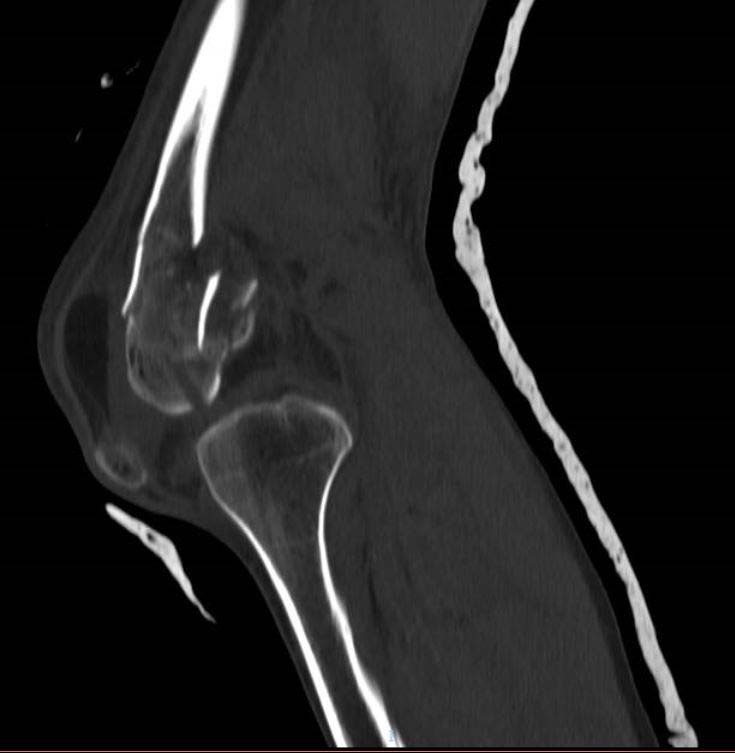
Use of CT Scan to determine fracture configuration, size of bony comminution and bone stock; Sagittal Section.

**Fig. (12):**
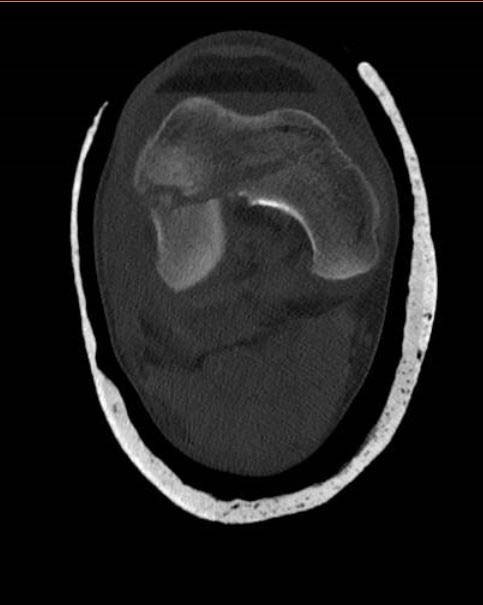
Use of CT Scan to determine fracture configuration, size of bony comminution and bone stock; Horizontal Section.

All distal femoral fractures with intra-articular comminution and extension should undergo a Computer Tomography (CT) scan with 2D and 3D reconstructions. This will readily identify a coronal plane fracture (Hoffa fracture) (Fig. **9**) that usually requires independent interfragmentary screw fixation. It also provides invaluable information on fracture configuration, size of bony comminution and bone stock (Figs. **10**, **11** and **12**) determining whether internal fixation is achievable. Current implant designs allow internal fixation in increasingly distal fracture configurations and primary distal femoral replacement is rarely performed.

### 4.7 Fixation Device

The AO/OTA classification mentioned previously highlights what fixation modality is required. Completely undisplaced 33-A1.1 fractures can be managed non-operatively with the affected limb placed in either a long leg cast or a hinged knee brace, however operative stabilisation is recommended to allow for early motion and rehabilitation. All other 33-A1, A2 and A3 fractures should be treated surgically unless the patient is medically unfit for an operation. For all 33-A fractures the option of closed reduction and minimally invasive submuscular fixation exists. Fixation devices in these circumstances are dynamic condylar screws (DCS), less invasive stabilization systems (LISS) or locked condylar plates (LCP) [24, 25]. Depending on the fracture pattern, retrograde IM nailing may also be used once closed reduction is achieved. In our experience, patients presenting with a ‘floating knee’ (concomitant ipsilateral tibial shaft fracture) should be managed with IM nailing as this allows access to both operation sites through a single incision. If an open approach is necessary, placement of a condylar locking compression plate or angled blade plate is standard practice. The use of these techniques in extra-articular distal femoral fractures has been well described in the literature previously [26].

**Fig. (13):**
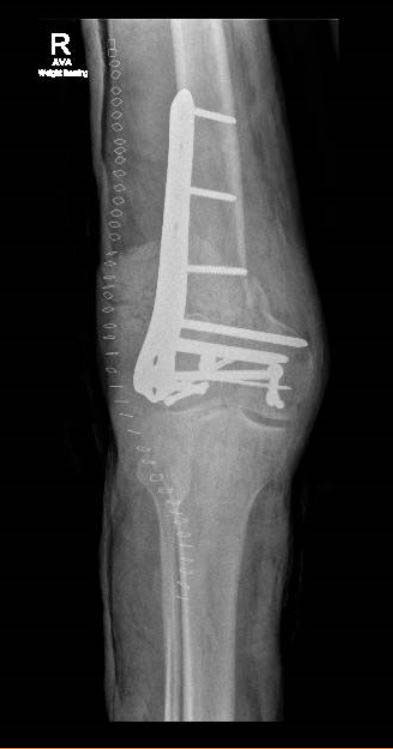
Use of multidirectional locking plate and multidirectional screws to achieve stable fixation; Anteroposterior Radiograph.

**Fig. (14):**
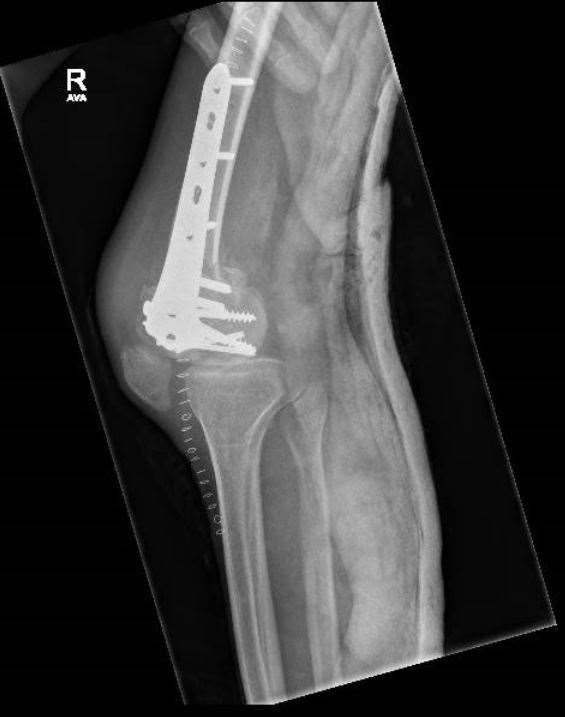
Use of multidirectional locking plate and multidirectional screws to achieve stable fixation; Lateral Radiograph.

For fractures with intra-articular extension (33-B and 33-C), open reduction and internal fixation with restoration of the articular surface and the relationship of the distal articular segment to the femoral shaft is recommended. Preference is for fixation utilizing an anatomically contoured lateral locking plate for angular stability. This offers a more biomechanically stable construct under physiological loading and is particularly helpful in osteoporotic bone [16]. A combination of partially threaded cancellous, cortical and headless compression screws are utilized in the metaphyseal portion to achieve articular congruity. Occasionally a headless compression screw buried in the articular cartilage is required in the sagittal plane to hold a coronal plane fracture. In newer generation multidirectional locking plates the locking screws can be directed through an arc of 20 degrees. This allows the surgeon to precisely direct screws thus avoiding metal conflict with other areas of distal fixation and thereby increasing stability with multi-planar fixation producing reliable results (Figs. **13** and **14**) [27].

### 4.8 Patient Position and Surgical Approach

Utilizing a radiolucent table that allows unobstructed fluroscopy of the whole femur is recommended. Place the patient in supine position with a bolster under the ipsilateral hip allowing the leg to lie in neutral rotation. Prepare and drape the entire leg. Use a radiolucent triangle under the thigh to help position the leg. Use a sterile tourniquet to allow access to the shaft of the femur. It is important to ensure unobstructed high quality AP and lateral imaging is achievable before starting the case.

Utilize a direct lateral approach for most intra-articular distal femoral fractures. Distally curve the incision anteriorly and incorporate a lateral parapatellar arthrotomy. The advantages of this approach include the ease of plate application, the ability to reduce the metaphyseal component and it’s extensile nature. To visualize the medial articular surface the approach can be extended distally to sublux the patella medially and the option to perform a tibial tubercle osteotomy can allow complete visualization of the articular surface in the complex 33-C2 and 33-C3 fractures.

### 4.9 Articular Reduction

In treating 33-C2 and 33-C3 fractures a pre-operative CT and adequate articular exposure is the key to accurate reduction. Indirect or percutaneous reduction and fixation techniques are not recommended because the goal of treatment is achieving anatomical reduction of the joint cartilage and stable fixation to allow early joint mobilization and rehabilitation. Hoffa fragments are addressed first by placing pointed reduction clamps perpendicularly across the fracture within the exposure. Upon achieving anatomical reduction the fragment is provisionally held with Kirschner wires (K-wires). At least two AP interfragmentary compression screws are used to control rotational forces and should be placed away from the weight bearing area of the articular cartilage. The screw heads must be countersunk below the cartilage surface to avoid impinging on the patella. Use variable pitch headless compression screws or countersunk partially threaded cancellous screws. Following fixation of Hoffa fractures, each condylar segment must be de-rotated and reduced using multiple k-wires or a pointed reduction clamp. Due to the trapezoidal nature of the distal femur and occasional loss of bone the reduction can appear anatomical at one point but mal-reduced at another point. Once anatomical reduction has been achieved, an interfragmentary compression screws should be inserted from lateral to medial to compress the femoral condyles. Care must be taken to place these screws anteriorly or posteriorly to avoid screw conflict with the distal locking screws of the plate.

### 4.10 Reduction of Articular Surface to Femoral Shaft

Once stable fixation of the articular segment has been achieved, it is reduced and fixed to the femoral shaft. In simple fracture patterns direct visualization with lag screw fixation or compression plating can be utilized. In more complex fracture patterns (AO 33-C2 and 33-C3) with metadiaphyseal comminution (Figs. **10** and **11**) the goals of treatment are to restore length, rotation and alignment of the distal articular surface to the shaft of the femur with minimal disruption to the fracture fragments. This reduces the risk of devascularisation, subsequent non-union and implant failure. Here a pre-contoured lateral distal femoral locking plate is used to bridge the area of metadiaphyseal comminution. Use a plate length that spans three times the length of comminution. In restoring the coronal alignment of the femur the pre-contoured plate can be useful once the plate is “reduced” onto the bone. Length, rotation and sagittal alignment can be more difficult to restore and is achieved through manual traction and manipulation of the distal segment with large diameter k-wires or a 5mm Schantz pin to joystick. A femoral distractor placed proximally in the shaft and into the distal articular block can be helpful to maintain traction, restore rotation and maintain sagittal alignment long enough to allow plate application to restore coronal alignment.

### 4.11 Post-Operative Management and Goals

As with all articular fractures, early range of motion and rehabilitation is key to preserving joint function. Therefore lower extremity range-of-motion exercises and gait training are advocated to commence on the first post-operative day. This rapidly progresses towards a physical therapy exercise program to improve knee range of motion and quadriceps function. Most patients are fitted with a hinge knee brace during the first 6 weeks of ambulation. For the complex 33-C2 and 33-C3 fractures, toe-touch weight bearing is recommended for 10 to 12 weeks. Sutures are removed at two weeks and patients are seen for clinical examination and radiographs at 6 weeks. Patients are radiographically assessed again at 12 weeks for evidence of callous and whether weight bearing can commence. Subsequent review is required every two to three months until clinical and radiographic union is achieved.

### 4.12 Limitations

The use of hospital coding records to derive cases for this study runs the risk of omissions. Incomplete electronic medical records can also affect elements of data collection. To ensure completeness of data capture, several different hospital databases were used to identify and independently confirm cases. As an unblinded observational study, there is risk of unintentional bias in interpreting radiographs. As mentioned previously, sex-based analysis was conducted in disaggregated data. There is the assumption that any differences between gender and biological sex are irrelevant in the context of sustaining distal femoral shaft fractures.

## CONCLUSION

This study adds to the existing body of knowledge regarding the epidemiology of distal femoral shaft fractures. It describes an injury that predominantly affects elderly women and younger men in a bimodal distribution. Sex and mechanism of injury are also closely correlated. Intra-articular involvement is common with differing degrees of complexity. Experience suggests the use of locking plates and IM nails are effective operative options. Due to low incidence, it is deemed appropriate to manage these fractures in specialized trauma centers.

## Figures and Tables

**Table 1:** Epidemiological Data for Patients Presenting with a Fractured Distal Femur.

	Whole Series	Male	Female	AO 33-A Overall	AO 33-A Male	AO 33-A Female	AO 33- B Overall	AO 33-B Male	AO 33-B Female	AO 33-C Overall	AO 33-C Male	AO 33-C Female
Mean Age (Yr)	63	61.6	64.0	64.6	62.4	66.5	60.2	57.9	63.2	63.1	71.3	56.3
Median Age (Yr)	67.5	65.6	71.0	71.2	66.2	72.6	65.2	65.1	65.1	63.3	85.7	55
Min Age (Yr)	15	15	17	17	17	17	15	25	15	21	21	27
Max Age (Yr)	101	92	101	101	91	101	95	92	95	91	91	86
Right Sided (%)	45.6, n=57	21.0, n=26	25.0, n=31	29.0, n=36	12.1, n=15	16.9, n=21	9.7, n=12	5.6, n=7	4.0, n=5	7.3, n=9	3.2, n=4	4.0, n=5
Left Sided (%)	52.8, n=66	21.0, n=26	32.3, n=40	21.8, n=27	4.8, n=6	16.9,n=21	21.0, n=26	11.3, n=14	9.7, n=12	10.5, n=13	4.8, n=6	5.6, n=7
Bilateral (%)	0.8, n=1	0	0.8, n=1	0.8, n=1	0	0.8, n=1	0	0	0	0	0	0
Associated Injury (%)	25.8, n=32	16.7, n=21	8.9, n=11	11.3, n=14	6.5, n=8	4.8, n=6	9.7, n=12	3.2, n=4	6.5, n=8	4.8, n=6	4.0, n=5	0.8, n=1
High- Energy Mechanism * (%)	45.8, n=44	32.3, n=31	13.5, n=13	17.7, n=17	11.5, n=11	2.3, n=6	16.7, n=16	12.5, n=12	4.2, n=4	11.5, n=11	7.3, n=7	4.2, n=4
Low- Energy Mechanism * (%)	54.2, n=52	9.4, n=9	44.8, n=43	33.3, n=32	31.3, n=30	2.1, n=2	13.5, n=13	5.2, n=5	8.3, n=8	7.3, n=7	2.1, n=2	5.2, n=5
Total Number of Patients	124	52	72	64	21	43	38	21	17	22	10	12

* High-Energy Mechanism comprises fall from height >2m, Road Traffic Collisions, Assault and Penetrating Injuries. Low-Energy Mechanism comprises of fall from<2m. Unspecified injuries (n=28) are excluded from this analysis.

## LIST OF ABBREVIATIONS

AO: Arbeitsgemeinschaft für Osteosynthesefragen
AP: Anterior-Posterior
CT: Computerized Tomography
DCS: Dynamic Condylar Screws
IM: Intramedullary
K-Wires: Kirschner wires
LCP: Locked condylar plates
LISS: Less invasive stabilization systems
MTC: Major Trauma Centre
OTA: The Orthopaedic Trauma Association Committee for Coding and Classification
SAGER: Sex and Gender Equity in Research
TARN: Trauma Audit and Research Network Registry
TKR: Total Knee Replacement
UK: United Kingdom
